# Learning From Self‐Care Practices Among Marginalised Women: Towards Embedding Health Justice in the UK Health Policy

**DOI:** 10.1111/1467-9566.70112

**Published:** 2025-10-23

**Authors:** Agata Pacho, Rebecca E. Glover

**Affiliations:** ^1^ Department of Health Services Research & Policy London School of Hygiene & Tropical Medicine London UK

**Keywords:** AMR, health policy, self‐care, urinary tract infections

## Abstract

Self‐care is a recurring theme in health policy, frequently positioned as a means of reducing demand on overstretched healthcare systems. In the United Kingdom, the growing interest in self‐care as a policy solution has been ascribed to the heightened prevalence of chronic illnesses, over 15 years of austerity and their interaction with neoliberalism. This article juxtaposes policy framing of self‐care with how it is conceptualised, practised and experienced by women from racialised minorities and low‐income households living in the United Kingdom—groups that routinely encounter structural barriers to healthcare access. This study centres marginalised communities, drawing on arguments that those in such positions hold epistemic advantage, as they often develop nuanced understandings of the social relations that shape their marginalisation. Through thematic analysis of focus groups and interviews, we identify two dominant ways women described and practised self‐care: (1) as a response to systemic care scarcity in healthcare services, workplaces and intimate relationships, and (2) as a relational practice embedded within informal networks of support. Grounded in an intersectional analysis, we argue these practices challenge dominant narratives of self‐care as an individualised responsibility. Recognising existing self‐care labour in marginalised communities requires health policies that address structural inequalities rather than shift burdens onto already constrained individuals.

## 1 Introduction

Richer countries with health systems struggling to provide quality, joined‐up care for their ageing populations increasingly advocate for ‘self‐care’. Often seen as a policy tool bridging the gap between supply of and demand for health services (Chapple and Rogers 1999; Wilkinson and Whitehead 2009), especially in publicly financed healthcare systems, self‐care is frequently described as an alternative to professional care and thus is assumed to make fewer demands on the budget (Redman 2007; Wilkinson and Whitehead 2009), while also being linked to notions of patient empowerment (see, e.g., World Health Organization 2024). Although self‐care lacks a single definition (Godfrey et al. 2011), it is commonly framed in terms of self‐management and autonomy, encompassing a wide range of activities from maintaining a healthy lifestyle and addressing emotional and psychological needs to managing chronic conditions and preventing illness (Barlow et al. 2002; British Medical Association 2022). In the United Kingdom (UK), self‐care has been an explicit feature of UK health policy for decades, including notably in New Labour's second term (2001–2005), which promoted patient choice and individual responsibility (Vizard and Obolenskaya 2013). Yet, the growing interest in self‐care as a health policy solution in recent years has been variously ascribed to the heightened prevalence of chronic illnesses (Wilkinson and Whitehead 2009), 15 years of austerity and the interaction between these and neoliberalism (Ward 2015).

Self‐care and terms like it have been included in antimicrobial resistance (AMR) policies and used to assert individual responsibility for preventing infections and managing symptoms of minor illnesses at home (see, e.g., Global and Public Health Group 2019). Yet, although AMR in the United Kingdom is a high‐priority policy area, the National Health Service (NHS) has not been funded over the last decade to cope with increasing populations, multiple comorbidities, poorer and sicker patients or, latterly, a shift of AMR policy away from acute hospital care towards community care. Meanwhile, patients are also people experiencing growing economic pressures due to the ‘cost‐of‐living crisis’, best defined as ‘a situation in which the cost of everyday essentials, such as groceries and bills, rises faster than average household incomes’ (Webster and Neal 2022; Brown 2002), which may limit their ability to take time off work, engage in self‐care and learn where to access care. Consequently, it has been argued that sustainable and impactful changes in antibiotic use can only be achieved by addressing the broader structural factors, such as austerity, that influence the poor health and insecure employment leading to antibiotic prescribing as state‐sanctioned economic infrastructure (Chandler 2019; Glover et al. 2023). Particular attention needs to be given to the specific challenges faced by marginalised populations, whose circumstances are shaped by intersecting forms of disadvantage (Adebisi and Ogunkola 2023).

Here, we draw on a study that examined the feasibility and appropriateness of the UK AMR National Action Plan (NAP)'s commitment to raising public awareness about the risks of antibiotic overuse by promoting self‐care for minor infections and reducing expectations of antibiotic prescriptions (Department of Health and Social Care 2019). We juxtapose how the UK policy frames self‐care as one of the solutions to growing AMR with how self‐care is already conceptualised, practised and experienced by women with recurring urinary tract infections (UTIs). We focus on groups facing significant barriers to healthcare—particularly women from racialised minorities and low‐income households.

### 1.1 Critique of Self‐Care: An Intersectional Perspective

Although self‐care is often presented in health policy as a means of improving patient autonomy and outcomes, critics have argued this framing reflects an over‐reliance on individual responsibility (Pyles 2020; Greaney and Flaherty 2020; Hafez et al. 2024). Rather than empowering, individualistic accounts of self‐care are applied in contexts marked by pre‐existing social and economic inequalities; such narratives risk overlooking the structural inequities shaping health outcomes (Pyles 2020).

Recently, the term self‐care has experienced widespread coverage across major online platforms, finding new resonance in popular culture (Kim and Schalk 2021). It has been argued that these new representations, often framed through a neoliberal lens, have promoted individualism and self‐reliance and are closely associated with consumerism (Martínez‐Jiménez 2022). In response, scholars have called for a return to the framing of self‐care long advocated by Black feminists, one that defines it not as an act of indulgence but of self‐preservation and community building (Kim and Schalk 2021; Martínez‐Jiménez 2022). Since the nineteenth century, Black feminist writers have linked self‐care to survival and social justice, putting forward a model of selfhood that is relational and grounded in solidarity (Rosenbaum and Talmor 2022). It has been theorised that as many Black women have been long subjected to domination, exclusion and erasure, self‐care could never be apolitical or opposed to collective action (Zuckerwise 2024).

Lorde (1988), a Black lesbian poet and theorist, described self‐care as a radical act of ‘self‐preservation’ and ‘political warfare’ (Lorde 1988). Scholars building on Lorde's work highlight the gendered, racialised and classed dynamics that shaped her thinking, using intersectionality to understand how multiple identities interconnect and influence experiences of care. Writing while living with cancer, Lorde did not view her illness as purely personal but as intertwined with ‘state‐sanctioned systems’ designed to control and exploit Black life (Lorde in Kim 2017). For those who are marginalised, then, Lorde's self‐care is not simply about maintaining or improving one's well‐being but a political act of defiance against a hostile and discriminatory environment.

Expanding on Lorde's work and drawing insights from queer of colour critique and disability theory, Kim (2017) suggests that for those facing structural oppression, self‐care is more often carried out in defiance of the state rather than as a result of government‐led guidance (Kim 2017). Further, self‐care, particularly when practised in response to systemic discrimination and oppression, does not equate to self‐sufficiency. Kim (2017) highlights the importance of networks of care, rejecting the ideal of individual self‐reliance. A similar critique is offered by The Care Collective (2020), a London‐based group formed to address ‘the multiple and extreme crises of care’, who criticise the neoliberal perspective on care being ‘up to the individual’ but neglecting to acknowledge ‘our shared vulnerabilities and interconnectedness’. This perspective, according to the Collective, while dominating current understanding of care, fosters a callous climate, particularly impacting those reliant on welfare and often trapping them in ‘worklessness and dependency’ (Chatzidakis et al. 2020, 12–13).

Tokumitsu’s (2018) critique of late capitalism similarly echoes the deep alienation and insecurity that neoliberal logic and the political economy have produced, while offering commodified and individualised self‐care as a form of relief (Tokumitsu 2018; Zuckerwise 2024). This contradiction mirrors what we observe in healthcare: As underfunded systems withdraw professional care, patients are encouraged to do more self‐care—some more than others.

Although we align with the existing critique of the neoliberal framing of self‐care, we also emphasise the importance of foregrounding the self‐care work already being undertaken by those who have been marginalised and underserved by healthcare systems. Here, we argue that recognising these efforts is essential to challenging narratives in health policy that place the burden of health management on individuals while failing to address systemic barriers to care.

### 1.2 Our Case Study—Self‐Care for Urinary Tract Infections

In this paper, we analyse how women experiencing symptoms of lower UTIs conceptualise and practise self‐care. Though often short‐term, UTIs can significantly impact quality of life, especially when recurrent (Witteman et al. 2021). Self‐care for UTIs recommended by the NHS includes hydration, over‐the‐counter (OTC) pain relief, cranberry‐based products and good hygiene (NHS 2022).

## 2 Methods and Methodology

### 2.1 Participants

The women in our study identified as belonging to racialised minorities and/or low‐income households, groups experiencing inequities in healthcare access and health outcomes despite the United Kingdom's nationalised system. Racial inequities persist, with Black women often facing harmful assumptions such as having higher pain thresholds (Peter and Wheeler 2022), contributing to the broader trend of women's pain being dismissed in clinical settings (Samulowitz et al. 2018; Hossain 2021). Racialised groups also experience poorer outcomes for chronic conditions, including recurrent UTIs (Jansåker et al. 2021). Economic precarity further limits self‐care. Those in insecure employment are less able to take time off to recover and may turn to antibiotics as a practical solution (Will 2018). Limited finances constrain access to OTC treatments, whereas basic needs (food, heating) often take precedence over health. These conditions intensify stress and make it harder to prioritise well‐being (Warner et al. 2021).

The aim of the sub‐study from which we here present findings was to understand the implications of AMR policy, particularly the way it promotes self‐care. We prioritised the perspectives of women who experience poorer access to essential health services and resources for self‐care. The decision to place women from marginalised communities at the centre of the analysis is rooted in feminist standpoint theory (Harding 2001) and is informed by Black scholarship (e.g., Baldwin 1963). These philosophical traditions highlight the socially situated nature of knowledge and posit that individuals occupying marginalised positions inherently possess an epistemic advantage, as they hold a more nuanced comprehension of the dynamics inherent in social relations and systems of power that contribute to their marginalisation than those in more advantaged positions. We thus argue that marginalised women's practice and conceptualisation of care is essential for identifying the shortcomings and gaps in the current deployment of self‐care within AMR policy.

### 2.2 Recruitment, Data Collection and Analysis

The study design and recruitment methods were shaped through discussions with a patient and public involvement and engagement (PPIE) panel of 10 participants who identified as women from racialised minorities, low‐income households or both. These meetings informed interview topics and optimal recruitment platforms. For example, we learnt not to ask interviewees where they live, but rather where they access NHS services, in case they do not have a permanent address or experience homelessness. We were also made aware of the healthcare access barriers our interviewees might face, which led us to dedicate more time in the interviews to discussing those issues. We invited PPIE members to take on co‐facilitation roles in focus groups and interviews. Although two members initially expressed interest, they later opted to remain in advisory positions, even though we had offered research methods training. This has prompted a rethinking of the wider research unit's PPIE strategy to ensure improved approaches and time allocated to training preparation and skill development.

Subsequently, this study was advertised via Call for Participants platform, social media, word of mouth and leafleting in two South London shopping centres. This led to four focus groups with 20 women, followed by in‐depth interviews with 14 participants. Additionally, five women were recruited after the focus groups, totalling 25 participants. Of these, eight had household incomes below £19k, seven identified as racialised minorities, and 10 belonged to both groups. Among women from racialised minorities, 14 identified as Black and three as South Asian. Most (76%) accessed NHS services in London. Women received £25 per participation.

Focus groups were led by AP, a White woman in her early 40s with over 10 years' qualitative research experience, assisted by a note‐taking colleague. AP introduced herself as a university researcher with no medical expertise on UTIs and no affiliation with policymakers or the NHS. Participants discussed their UTI history, advice‐seeking behaviours, self‐care practices and the broader role of self‐care in their lives.

Focus groups provided insights into shared experiences, whereas semi‐structured interviews allowed for more personal reflections. Most participants kept cameras off during the focus group discussions, fostering anonymity and candidness (Alliance Scotland 2019). However, the lack of visual cues required a more active facilitation approach. The dynamic resembled one‐on‐one interviews, with participants sharing experiences equally openly. As a result, findings from both methods were similar and are presented together.

The focus group discussions and interviews were transcribed verbatim. In addition, the researcher took notes following the focus groups and interviews. On one occasion, we quote directly from notes where the interviewee made it clear that she had forgotten to mention the detail in the interview. The transcripts were analysed thematically (Riger and Sigurvinsdottir 2016). We first identified themes and assigned descriptive codes, grouping together those that captured similar topics to move from descriptive themes to more analytical categories. For instance, codes such as ‘delayed GP appointments’ and ‘unable to call in sick’ were interpreted as indicators of the scarcity of care in women’s lives. The analytical approach draws on theoretical conceptualisations from the critique of the neoliberal framing of self‐care that highlights the gendered, racialised and classed dynamics that influence experiences of care.

This study was reviewed and approved by the Health Research Authority (REC Ref: 22/HRA/3073) and the London School of Hygiene and Tropical Medicine Ethics Committee (LSHTM Ethics Ref: 27930). To protect anonymity, all names used in this article are pseudonyms.

## 3 Findings

We identified two key themes reflecting how the interviewed women practised and conceptualised self‐care: self‐care as a response to the systemic lack of care (including professional healthcare, workplace support and care within intimate relationships) and self‐care as a collective practice.

### 3.1 Self‐Care as a Response to Systemic Scarcity of Care

#### 3.1.1 Scarcity of Professional Healthcare

All women that took part in our research had been diagnosed with a UTI at least once. Most reported recurring UTIs with symptoms including increased urgency to urinate, pain when urinating and an ‘unpleasant’ smell. Although most women were familiar with NHS guidance on self‐care practices for UTIs, they often relied on these strategies not solely by choice but due to broader barriers in accessing healthcare services. Frequently, they regarded the practice of self‐care as their initial action when they could not book an appointment with a general practitioner (GP). This is in line with research conducted in the United Kingdom that people prefer to look after minor illnesses themselves or visit a pharmacy (O’Cathain et al. 2020). One woman who identified as South Asian and from a low‐income household shared that she struggled to access primary care and believed that the ongoing difficulty in securing timely GP appointments was a lingering effect of the Covid‐19 pandemic's disruption to the health system. Discouraged by past experiences, she began adopting a more self‐reliant approach, actively avoiding healthcare services:

(…) it was painful and everything, yeah, and I had to go to the GP basically. I tried to avoid going because that time it was very difficult to book like face‐to‐face appointments (…) And then eventually when I just couldn’t take it anymore, my last resort, I had to just basically go to the GP and speak to my doctor.

(Angela)

Another woman, a White immigrant living in a low‐income household, told us how she had to prepare herself for an ‘uphill battle’ whenever she needed to access healthcare, including for the diagnosis and treatment of recurring UTIs. She suspected that part of this difficulty stemmed from being an immigrant, in particular being unfamiliar with the UK healthcare system and speaking with a non‐British accent:

(…) I think very weirdly this was another reason why I didn’t get access to proper healthcare (…) You’re dealing with a person who doesn’t understand you fully, I used to have a stronger accent at the time. And they make you run through circles in which you do not understand what they are saying because they’re speaking extremely fast and you do not understand the medical system under which they’re operating.

(Irina)

Irina has lived in the United Kingdom for several years and noted that her experience has changed as her accent has become less pronounced and her English has improved. Her reflections underscore the layered vulnerability experienced by migrants; healthcare access can be obstructed not just by logistical or bureaucratic hurdles, but by the emotional toll of having to rely on one's non‐native language.

Even when language was not a barrier, the UK healthcare system could still appear less accessible than those in women's countries of origin. Anika, a South Asian migrant and university student fluent in English, found UK healthcare services frustrating, particularly the rigid appointment system:

Yes, so maybe it’s a slightly cultural thing, I come from a country where people are always around you, and there’s so many people, you know you can call the doctor and they’d be like, “Yes of course we’re here”, and you can walk into a doctor’s clinic at any time, and there will be someone to help you. I think this is also quite a foreign concept that I need to call a receptionist who will give me an appointment for the next day for a doctor who will call me at a specific 15‐minute time slot.

(Anika)

As migrant women, Anika and Irina expressed a shared sense of otherness in relation to the UK healthcare system. In both cases, they faced delays in diagnosis and treatment. Previous research has highlighted how social and economic inequalities often push those who are marginalised and have restricted access to professional care to rely more heavily on self‐care (Hafez et al. 2024); such overreliance on self‐care places them at greater risk of poorer health outcomes (Greaney and Flaherty 2020).

Other women described hesitancy about contacting healthcare professionals, even when symptoms were uncomfortable and recurring. Emma, who identified as South Asian, began experiencing UTIs as a student, shortly after becoming sexually active with her then‐boyfriend. She said she quickly linked her symptoms to sexual activity. Although Emma said that made her feel too embarrassed to seek professional care, she was also uncertain whether the symptoms justified seeking help:

I think I was a bit embarrassed about going to see a doctor and although I was very uncomfortable with the symptoms, it just didn’t feel like it was serious enough to go and try and get a doctor’s appointment. So, similarly, I looked online, I sort of figured out what it was, and it was on the NHS website. I also drank tons of water, went to the pharmacy and got the over‐the‐counter sachets and managed it that way.

(Emma)

Later in the interview, Emma explained that she only discovered how to prevent recurrent UTIs by chance when a friend mentioned that his partner stopped experiencing them after urinating immediately after intercourse. Ultimately, Emma managed her recurring condition through self‐care, relying on information from the internet and learning an effective prevention method from a friend. All the while, she assumed that her condition was not ‘serious enough’ to warrant seeking professional care.

Hesitancy or embarrassment about contacting healthcare professionals for UTI‐related care emerged as a consistent theme. Women often described the symptoms as ‘intimate’ or even ‘dirty’, drawing our attention to the stigma surrounding intimate health and well‐being:

I was afraid of being judged because, you know, the smell, it’s a bit uncomfortable. So when you have that stinking smell, I was afraid of people judging me, yes.

(Ayanda)

And the first GP that I contacted was a male GP and it wasn’t easy actually explaining to me all that I was going through but I tried. (…) I kept on like searching for a better person that I could trust with my problems and issues (…) I got a female GP this time around and she’ll be of great help, she’ll be helping me.

(Sade)

Women who struggled to seek professional care or discuss their symptoms with their GPs appeared to assume that their concerns would not be met with a safe and nonjudgemental response. They referenced the stigma they perceived around symptoms affecting intimate areas, which discouraged them from seeking medical support, a barrier already identified in UK primary care (Hurtaud et al. 2025).

The above quotes also illustrate how women engaged in self‐triaging. They made decisions about when to enter professional healthcare in response to structural barriers (e.g., limited access to care), they weighed symptom severity against social risks of censure, and they undertook the labour of finding a healthcare provider with whom they felt safe. Self‐triaging may, therefore, be an additional component of self‐care and one that does not result from the NHS guidance for UTIs but, rather, is a result of the inaccessibility of the healthcare services. This reflects arguments that individuals with limited resources often engage in self‐care irrespective of government (Kim 2017) and that such practices can be labour‐intensive (Greaney and Flaherty 2020).

Another interviewee, a White woman who lived in a low‐income household, pointed out that when seeking professional healthcare, women tend to face delays due to being passed between services without receiving adequate treatment:

[It’s] on my mind at the minute speaking with one of my friends, because she’s currently just been taken to hospital for an infection and kind of has been trying to get an appointment with doctors and trying to get an emergency appointment. And it turned out not to be a UTI but everyone on the phone and 111, the GPs, the sexual health clinics, were all kind of telling her just not to use perfumed soap and to use like hot compresses and stuff like that. And it’s been like a main topic with all me and my friends at the minute because of that, and because of those kind of like that advice and the kind of not having taken her seriously and kind of women’s health seriously.

(Elizabeth)

Experiencing her first UTI as a 6‐year‐old, now in her 20s, Elizabeth described herself as having a good knowledge of her body and UTIs and being comfortable talking about them with others. Her account highlights her awareness that women are discriminated against within the healthcare system when women are not recognised as credible knowers of their own bodies and their symptoms are being trivialised, aligning with established research (Samulowitz et al. 2018; Hossain 2021). The scarcity of care within the NHS was perceived as a shared experience, with women attributing it to widespread sexism within healthcare services, manifesting as inadequate health education surrounding important yet overlooked health concerns outside the realm of reproductive health. For example, many women in our study admitted they had little awareness of UTIs and their prevalence before being diagnosed:

I feel like that should be part of some sort of health education alongside, you know, we talk to girls about menstruation which affects almost all women. (…) Obviously, it was a long time since I was at school and I’m sure they talk about lots of other things like consent and HPV and other stuff. Yes, I think if that had even been mentioned once, that probably would have been somewhere in my brain because it’s the kind of thing that I would—you know, I wanted to look after myself probably and I didn’t know what I was doing.

(Emma)

The gap in medical knowledge and attention to women's health beyond reproductive issues, brought to our attention, has been well documented (e.g., Peters et al. 2016). Another woman confided post‐interview about her intention to establish a local initiative aimed at educating women about UTIs. She aspired to bridge an educational gap she observed while connecting with others in the community who shared her experience of recurrent UTIs. Her initiative, in the face of care scarcity, could be seen as a form of community‐based self‐care.

#### 3.1.2 Scarcity of Care: Workplace

Women also highlighted the scarcity of opportunity for self‐care available to them in workplaces. Those able to take time off work to recover from UTI episodes expressed appreciation for their line managers, whom they described as uniquely understanding. Among those living in low‐income households, two women working as teachers highlighted how their specific work environment and job demands not only made it difficult to take time off for recovery but also hindered their ability to prevent UTIs in the first place.

(…) when you work in a school and you work with kids, it’s really hard to go to the toilet regularly. And I think one of the one things that does worry me and has become more of a preventative thing that I’ve tried to be better at is actually going to the toilet when I need to go, rather than just holding it for ages. (…) I think lots of teachers especially really struggle with this.

(Eva)

For me I just try and get on with my like daily life as much as possible, so that includes going to work. I don’t feel able to—I wouldn’t feel able to call in sick to work with a UTI. So if there was more understanding perhaps at work it would be nice.

(Celine)

On the other hand, Elizabeth was a student based in London who also worked freelance to support herself. She explained how she had to negotiate what she was able to cancel and when she had to push through with professional commitments, even when in pain. Paid work, in particular, was often non‐negotiable, as it was her primary means of financial support:

I think I’ve had to miss a few lectures, I think I have had a few times where I just had like really serious work days, like in terms of freelance stuff, like had things that I have to go to and I know that I have to go to them, so I’ve like not missed them, which has been just like really painful.

(Elizabeth)

Elizabeth's account highlights how access to rest and recovery is shaped by economic precarity and the demands of self‐employment. Although the university allowed for occasional absence, paid freelance work remained largely inflexible. Her narrative underscores the embodied consequences of gig economy labour, where care for oneself often comes second to the need to earn a living.

Although health policy and public health guidance often frame self‐care as widely accessible and enhancing patient autonomy, they ignore the fact that the ability to practise self‐care is deeply shaped by labour conditions and socioeconomic position (Pyles 2020; Greaney and Flaherty 2020). On the other hand, accounts shared by women in our study reveal economic barriers to self‐care faced by those whose work is undervalued or in precarious forms of work, underscoring what has been described as a feature of neoliberal economies, where ‘growing the economy’ is prioritised over the well‐being of citizens (Chatzidakis et al. 2020).

#### 3.1.3 Scarcity of Care: Relationships

Women also encountered a lack of care in their relationships with men. Elizabeth, introduced above, shared that her boyfriend struggled to comprehend UTIs, failing to understand why she appeared ‘outrageously grumpy’ and in ‘desperate need’ of assistance during symptomatic episodes. Elizabeth attributed this lack of empathy to being in a relationship with a cisgender man, implying that her experiences were, to some extent, expected in that context.

Another participant, a Black migrant woman living in London with her husband and two children, recounted a distressing experience where the symptoms of UTIs placed her at risk of partner violence and potential divorce. She described how her husband, whom she described as being violent towards her in the past, used the situation to intimidate her, because symptoms of UTIs can be similar to those of sexually transmitted infections (STIs):

So I had to tell him [the husband] what I was going through because it would be like every time I want to go to the toilet I was hesitating (…) So he noticed and asked me, “What’s happening?” And I didn't want to tell him, but I had to. I told him this is what’s happening, I’m feeling this way and this way, and he was like, “Have you started dating those men who bring products to you?” And I was like no, because he had some trust—OK, that’s family matters, we have some trust issues. So he was like, “Have you started cheating on me? Should we get my lawyer and get divorced?”

(Ayoka)

Ayoka shared that although her primary role was caring for her family, she had recently started a small business to contribute financially. Her husband, who travelled for work and was frequently absent, had grown increasingly jealous of the new relationships she had formed through work. When Ayoka told her GP about her husband's threats, the GP arranged a mediated disclosure to explain her UTI diagnosis and clarify that UTIs are not sexually transmitted, supporting her safety. From Ayoka's perspective, living with her husband made it difficult to hide health issues but unsafe to disclose them. Gendered violence in this instance—though part of a pattern of abuse—was compounded by the ambiguity of UTI symptoms, showing how illness can be co‐opted into forms of coercive control.

Ayoka’s and Elizabeth's stories suggest that being a woman in a heterosexual relationship could render relatively minor illnesses, such as UTIs, more distressing. Further, their accounts indicate that some women may practise self‐care within environments of emotional insecurity or outright risk. The narratives shared by women in our study demonstrate how women's pain is deprioritised both in relationships and in healthcare services. Consequently, self‐care, practised in the face of scarcity of care within the formal health system, in workplaces and in relationships, is inextricably tied to women's systemic experiences of marginalisation. Although self‐care has been recognised as both a radical act of ‘self‐preservation’ and a political act of defiance against hostile states (Lorde 1988; Kim 2017), the oppression women face within their homes and intimate relationships complicates its practice. When self‐care places women at risk if enacted openly, it no longer aligns with the notion of self‐preservation. The analysis, therefore, requires attentiveness to contexts in which self‐care exposes women to danger, while also recognising, echoing Lorde, that intimate and domestic violence is inseparable from the broader structures of sexism and heteronormativity (Lorde 1984).

### 3.2 Collective Self‐Care: With Others and for Others

To understand how to manage UTI symptoms, women turned to their informal support networks. They sought guidance from women in their families and trusted female friends who had first‐hand experiences with UTIs. Self‐care emerged as deeply embedded in friendships, familial relationships and collective forms of knowledge‐sharing. Finding reassurance, guidance and emotional validation outside of formal medical spaces was particularly important to those who felt hesitant about discussing UTI symptoms with their doctor. One of the women, Tamara, a Black woman living in a low‐income household, explained how she initially mistook her symptoms for an STI and felt nervous about speaking to her GP, whom she would usually contact for health advice. She found it easier to confide in her friend:

I talked to a friend who had experienced something similar, and yes, she advised me that it’s not good to ignore the symptoms (…) And she advised me, if I don’t see a specialist at that moment, it might even lead to more serious problems.

(Tamara)

Although we have shown earlier how the perceived stigma surrounding symptoms affecting intimate areas often discouraged women from seeking medical help, Tamara's account suggests that informal support networks can play a vital role in managing that stigma and enabling women to overcome barriers to care. In this context, trust, familiarity and embodied experience may complement medical expertise and, in some cases, serve as a necessary precursor to accessing formal care. This contrasts with policy narratives that frame self‐care as an individualised activity that can substitute for professional care (Barlow et al. 2002; British Medical Association 2022).

Support from a friend was also crucial to Ayanda, a Black woman dealing with UTI symptoms at the time of the focus group and who was in recovery by the time of the interview. She disclosed that the UTI had resulted from sexual assault. Ayanda described feeling ‘scared’ and ‘uncomfortable’ about seeking help from a healthcare professional at this particularly difficult time. Her friend reassured her that medical attention was necessary to prevent the symptoms from worsening, booked an appointment for her and accompanied her during medical consultations:

(…) She helped me with whatever. Anything that I needed in that moment, it was a good experience because she was there for me and she was guiding me. Even though we are not the doctors to deal with it properly, but she helped me and I was grateful for that. (…) I was a bit scared to go to the doctor, then you tell him you have this infection.

(Ayanda)

Ayanda also explained how her friend made sure she took her antibiotics on time. For her, care was enacted collaboratively, which seemed necessary and beneficial.

Close networks also enabled the kind of embodied care needed during periods of acute discomfort, including practical assistance such as sharing household duties. When, during one of the focus groups, another participant said they tried to carry on with their usual responsibilities while experiencing UTI symptoms, Sade objected:

As for me, I think it would be better if you have that close person who understands you better. I live with my sister who is—she knows everything that I go through. She knows whatever I’m suffering from and so when this condition is too worse for me to withstand, she’s always there taking good care of me, even carrying on with my duties

(Sade)

Indeed, the ability to seek help and support from others, including healthcare professionals, was perceived as bringing a sense of greater agency and deemed an essential aspect of self‐care. Anika, introduced earlier, explicitly redefined self‐care as the capacity to reach out and receive support when needed:

To me, I think self‐care is a lot more holistic in terms of—I think in terms of what you do for yourself, and how you make yourself feel looked after, but also that you’re able to reach out for help when you need it. I think that’s also a part of self‐care in being able to seek help, and get it when you need it the most

(Anika)

Self‐care was not only undertaken with support from others but was also understood as having protective value for others, particularly within the family. Ayoka, for instance, described her life as centred around her husband and two children. As the primary caregiver, she saw her own well‐being as closely linked to the well‐being of her family. Self‐care, then, was not only a collective act in how it was received but also in how it was practised for the benefit of others:

Self‐care, I guess it is me taking care of myself, and when I take care of myself, other people are as well protected because I’ll ensure everything that I am doing is hygienic. If I’m cooking food, the food is very clean. Whatever I’m using is clean. So meaning my family will not have unhygienic things or eat food that is not hygienically prepared (…) I’ll make sure that no one will not suffer (…)

(Ayoka)

In other words, responsibility towards others appeared as inherent to self‐care. Rather than resulting from policies that may ascribe individuals with blame, self‐care may follow on from an awareness of the importance of interdependence.

Responsibilities enacted through relations of care constitute a key form of responsibility today, and ‘competing responsibilities’ are inherent in contemporary social life (Trnka and Trundle 2014). The women in our study illustrated both moments where various care responsibilities aligned—such as when, for Ayoka, caring for oneself also safeguarded the well‐being of their family—and instances of competing obligations, for example, when tensions emerged between the need for self‐care and their professional responsibilities as teachers for Eva and Celine.

Moreover, forms of collective self‐care were not equally available. Access to informal support networks was shaped by broader structural factors, including migration status, which contributed to the precarity. For Abi, a Black woman living in a low‐income household, the absence of a reliable care network underscored the fragility of informal systems, especially in diasporic contexts:

Well, for me, in our own way, we all have our lives and being an African who is still trying to survive somewhere which is not at home—I don’t know if you are understanding? Everyone is busy. Like, no‐one has time. So you have to do your thing. So I think it’s hard. It’s fairly hard when you have no one.

(Abi)

This quote reminds us that collective support for self‐care is not universally accessible and, therefore, must be understood within intersecting inequalities, including racialisation and migration, that shape who is able to rely on others and in what ways. Although some participants resisted individualising discourses by emphasising interdependence, Abi was left to navigate illness in relative isolation. It is important to linger here—the burdens and benefits of care are unequally shared (Lynch 2009). In environments that promote hyper‐individualisation and alienation (Tokumitsu 2018), those who are marginalised by race, class or migration status may be at greater risk of exclusion.

## 4 Discussion

Self‐care was conceptualised, practised and experienced by women in our study (1) in the context of care scarcity within discriminatory systems and environments and (2) supported by—and undertaken for the benefit of—others. The way self‐care was practised and understood by the women in our study, therefore, required far more resources than those outlined in the NHS’s prescriptive, medicalised guidelines and definitions, which primarily focus on symptom management. Although the UK AMR policy asserts individual responsibility for preventing infections and managing symptoms of minor illnesses at home (Global and Public Health Group 2019; Department of Health and Social Care 2019), our findings demonstrate that women are already actively assuming responsibility for their own health and the well‐being of others.

However, echoing critiques of self‐care from an intersectional perspective (e.g., Lorde 1988; Kim 2017), this care was not a result of government‐led guidance. In fact, women's self‐care practices have revealed a fundamental disconnect between how the patient is conceptualised within the policy—as needing to be encouraged to—and how self‐care is actually enacted. Women's self‐care practices reflect a parallel reality shaped by structural constraints, care scarcity and everyday forms of marginalisation. Moreover, they performed self‐care alongside other care responsibilities, including family obligations and professional caregiving roles.

In the discussion that follows, we critically examine the relationship between care responsibilities and opportunities for self‐care to interrogate how this interdependence may shape and constrain women's well‐being. We then propose a reconceptualisation of the patient that centres social justice within health policy to shift responsibility away from the individual and calls for investment in the collective well‐being of marginalised groups.

### 4.1 Who Provides Care and Who Gets to Self‐Care?

Women in our study consistently turned to networks of family members and friends to manage UTI symptoms and to navigate barriers to healthcare. In some cases, informal support was a crucial precursor to accessing formal care. For those most reliant on such networks, informal care felt more tangible and dependable than the formal healthcare system itself. This logic of interdependence stands in sharp contrast to dominant health policy discourses, which typically frame self‐care as a tool for individual empowerment and personal responsibility. Instead of aligning with the neoliberal ideal of the ‘autonomous, entrepreneurial and endlessly resilient’ citizen (Chatzidakis et al. 2020), the self‐care practices described by our participants reframe care as mutual, relational and embedded in shared vulnerability rather than individualism.

Yet, it is important to resist romanticising this interconnectedness. Collective support for self‐care was not accessible to all women in our study, and our findings suggest that it must be understood in the context of intersecting inequalities, including racialisation and migration status, that shape who can rely on others and how. Further, we learnt that women undertook self‐care while also managing a range of other care responsibilities, some of which were competing. The coexistence of these obligations highlights the deeply interdependent and often gendered nature of women's lives. The gender inequalities in the distribution of care responsibilities have been well documented (Lynch 2009; Ehrenreich and Hochschild 2003; Bettio and Plantenga 2004; McGinnity et al. 2005), and Lynch (2009) has conceptualised this as affective inequality—where the burdens and benefits of care are unequally shared (Lynch 2009). Moreover, historically, Black women have often been cast in the role of caretakers within both families and communities (Hill Collins 2000 in Wyatt and Ampadu 2022) and seen as social activist leaders (Addai 2020 in Wyatt and Ampadu 2022). These roles can hinder their ability to prioritise their own self‐care (Wyatt and Ampadu 2022). In addition, The Care Collective (2020) points out:

(…) for women with slightly more resources, relieving the double burden [of paid professional work and unpaid domestic labour] has meant employing other women, predominantly poor, immigrant, and non‐white women to shoulder the bulk of caring labour, particularly domestic servicing. This has in turn facilitated exploitative transnational care chains where women from the global South migrate to the Global North to find jobs as care workers, often leaving their own children to be looked after by others. Racism thus combines with gender and global inequality to devalue the labour of care, ensuring the low pay and frequent exploitation of so many care workers, however essential and precious their caring labour is to their employers.

(The Care Collective 2020: 25–26)

In other words, the very expectations of care and resilience placed on marginalised women may themselves become barriers to meeting their own needs. Understanding self‐care through this lens reveals how the opportunity to care for oneself is unequally distributed and embedded within broader systems of structural exploitation.

### 4.2 Embedding Intersectionality and Social Justice in Health Policy

Far from being a sign of policy success, the self‐care work undertaken by women in our study reflects resilience in the face of systemic failure and limited resources. When health policy frames self‐care as a tool for antimicrobial stewardship, it obscures the social and structural conditions that shape how, and for whom, it is possible. On the other hand, recognising the existing, often invisible self‐care efforts of marginalised populations opens space to reconceptualise the patient. By ‘patient’, we mean anyone who accesses healthcare, either through consultations with healthcare professionals or through self‐care practices to manage illness, for example, using OTC medications or simply taking time to rest. In this way, we acknowledge the forms of healthcare that people provide for themselves when experiencing symptoms, as well as the work identified in this study that may precede formal healthcare use, such as self‐triaging and navigating complex health systems. We argue that, rather than seeing the patient as an autonomous individual detached from context, they can be understood as embedded in systems of inequality and interdependence.

Further, the health justice framework offers a productive lens for reconceptualisation of self‐care. Wiley et al. (2022) argue that health justice centres social justice as a foundational value of health policy, demanding that attention be paid to systemic oppressions and calling for critical scrutiny of how racism, classism and other structural inequalities shape the design and implementation of ostensibly equitable health measures (Wiley et al. 2022). Applying this framework to self‐care reframes it not as a policy instrument for individual behaviour change, but as a relational and structural issue. This approach aligns with Lorde's (1988) radical conception of self‐care, which foregrounds its racialised, gendered and classed dimensions (Lorde 1988). It shifts responsibility away from the individual and towards the state and broader society, requiring investment in the collective well‐being of marginalised groups. Within this framing, self‐care becomes a right enabled by equitable access to care, resources and safety, rather than a burden borne in the absence of adequate professional healthcare provision.

Bowleg (2012) argues that a commitment to social justice in health policy can be strengthened by employing intersectionality as an analytical framework by offering a multidimensional lens that reflects the complex realities of those most affected by health disparities (Bowleg 2012). The women in our study shared experiences shaped by interlocking systems of oppression, including racialisation and economic precarity. Their accounts did not merely illustrate the structural barriers they encountered but also offered critical insights into the limitations of current self‐care health policies. Their narratives highlight the disconnect between how responsibility for health is conceptualised in policy and how it is lived and negotiated in everyday practice. Drawing on intersectionality enables a more nuanced understanding of self‐care that foregrounds social justice.

## 5 Limitations and Mitigation

Given that most of our participants lived in London, our ability to comment on potential variations in accessing professional health services for UTIs and resources for self‐care in other regions of the United Kingdom, particularly rural areas, is constrained.

We chose to conduct focus groups online to reduce participation barriers for low‐income and minority groups by removing the need for expenses such as transport and childcare (Watkinson et al. 2021). Although these costs could be reimbursed, participants would have to cover them upfront per the conditions of our grant and manage the additional logistical burden of arranging childcare. However, the online recruitment methods and conducting focus groups online could have constrained participation for those with no access to the required technologies.

Further, our findings indicate minimal disparities between women belonging to racialised minority groups and those residing in low‐income households. Existing research highlights the distinct challenges faced by individuals from racialised minorities in accessing health services (Watkinson et al. 2021). Consequently, it is plausible that participants in our study may have hesitated to voice such concerns in the presence of a White interviewer. We acknowledge our positionality and the potential limitations it may have placed on our understanding of interviewees' experiences. To mitigate the risk of misreporting, we held a meeting with the PPIE panel to present the initial findings and gather their perspectives. Although their impressions did not differ from ours, the discussion reinforced our sense of how problematic the self‐care policy may be.

## 6 Conclusion

We have demonstrated how women experiencing recurrent UTIs, particularly those from racialised and low‐income households, are already engaged in extensive self‐care practices, often as a response to systemic failures in healthcare. We have highlighted a critical disjuncture between top‐down notions of responsibility embedded in health policy and the realities of care under structural constraints. Self‐care, as enacted by our participants, is relational, interdependent and shaped by unequal access to resources. Applying a health justice lens further reveals the limitations of framing self‐care as an individual responsibility and underscores the need for policies that account for social inequalities. Reconceptualising self‐care as a right, and not a substitute for professional care, requires structural support and investment in the well‐being of marginalised communities, rather than further responsibilisation of those already navigating care scarcity.

## Author Contributions

**Agata Pacho:** conceptualization (lead), investigation (lead), data curation (lead), formal analysis (lead), methodology, writing – original draft preparation, writing – review and editing (equal). **Rebecca E. Glover:** conceptualization (supporting), data curation (supporting), formal analysis (supporting), project administration (lead), supervision (lead), writing – review and editing (equal).

## Ethics Statement

This study was reviewed and approved by the Health Research Authority (REC Ref: 22/HRA/3073) and the London School of Hygiene and Tropical Medicine Ethics Committee (LSHTM Ethics Ref: 27930).

## Consent

Informed consent was obtained from all participants.

## Conflicts of Interest

The authors declare no conflict of interest.

## Permission to Reproduce Material From Other Sources

The authors have nothing to report.

## Data Availability

The authors have nothing to report.
